# Does the “Blue Sky Defense War Policy” Paint the Sky Blue?—A Case Study of Beijing–Tianjin–Hebei Region, China

**DOI:** 10.3390/ijerph182312397

**Published:** 2021-11-25

**Authors:** Xuan Yang, Yue Wang, Di Chen, Xue Tan, Xue Tian, Lei Shi

**Affiliations:** 1 School of Environment and Natural Resources, Renmin University of China, Beijing 100872, China; yangxruc@126.com (X.Y.); wangyue_prc@ruc.edu.cn (Y.W.); chendi16@ruc.edu.cn (D.C.); 2017200676@ruc.edu.cn (X.T.); 2 Energy Strategy and Planning Research Department, State Grid Energy Research Institute Co., Ltd., Beijing 102209, China; tanxue@sgeri.sgcc.com.cn

**Keywords:** blue sky defense war policy, monitoring point, air pollution, meteorological factors, difference-in-difference, quasi-natural experiment

## Abstract

Improving air quality is an urgent task for the Beijing–Tianjin–Hebei (BTH) region in China. In 2018, utilizing 365 days’ daily concentration data of six air pollutants (including PM_2.5_, PM_10_, SO_2_, NO_2_, CO and O_3_) at 947 air quality grid monitoring points of 13 cities in the BTH region and controlling the meteorological factors, this paper takes the implementation of the Blue Sky Defense War (BSDW) policy as a quasi-natural experiment to examine the emission reduction effect of the policy in the BTH region by applying the difference-in-difference method. Results show that the policy leads to the significant reduction of the daily average concentration of PM_2.5_, PM_10_, SO_2_, O_3_ by −1.951 μg/m^3^, −3.872 μg/m^3^, −1.902 μg/m^3^, −7.882 μg/m^3^ and CO by −0.014 mg/m^3^, respectively. The results of the robustness test support the aforementioned conclusions. However, this paper finds that the concentration of NO_2_ increases significantly (1.865 μg/m^3^). In winter heating seasons, the concentration of SO_2_, CO and O_3_ decrease but PM_2.5_, PM_10_ and NO_2_ increase significantly. Besides, resource intensive cities, non-key environmental protection cities and cities in the north of the region have great potential for air pollutant emission reduction. Finally, policy suggestions are recommended; these include setting specific goals at the city level, incorporating more cities into the list of key environmental protection cities, refining the concrete indicators of domestic solid fuel, and encouraging and enforcing clean heating diffusion.

## 1. Introduction

Since the reform was launched in 1978, China has experienced rapid economic and social development; on average, over 9% of GDP growth has helped China become one of the biggest economies [1,2]. However, extensive developments have led to environmental predicaments [3], especially increasingly severe air pollution, which has aroused significant concerns from the public and the government [4,5]. In 2017, 70.137% of all 365 cities’ ambient air quality exceeded the World Health Organization (WHO) Interim Target-1 (IT-1) of 35 μg/m^3^ [6].

Air pollution is a major contributor to the global burden of disease [7], including cerebrovascular diseases [8], respiratory diseases [9], lung cancer [10] and mortality problems [11]. In China, about 1.2 million people died prematurely due to complication associated with air pollution in 2010 [12]. In 2019, it is estimated that 12% of all deaths were attributable to outdoor and household air pollution [13]. In view of the scale of the impacts of air pollution on public health [14], urgent action is needed to improve air quality. Air pollution has also become a central domain and a momentous bottleneck in realizing the sustainable development in China [15].

China’s government has urged that the reduction of PM_2.5_ and other pollutants in smoggy cities is the key task in the fight against air pollution [16,17]. In September 2013, the State Council issued the “Action Plan for the Prevention and Control of Air Pollution”, which included ten specific measures and set specific PM_2.5_ reduction targets [18]. In March 2017, the Ministry of Ecology and Environment (the former the Ministry of Environmental Protection) issued the “Work Plan for Air Pollution Prevention and Control in Beijing, Tianjin, and Hebei and Surrounding Areas” [19], which determined the air pollution transmission channel cities in Beijing-Tianjin-Hebei (BTH) region and formulated the target to improve the air quality of northern China [20]. In July 2018, the Blue Sky Defense War (BSDW) policy was promulgated [21]. Compared with previous air pollution control policies, the BSDW policy highlights precise strategy implementation, pollution source control and long-term mechanism construction.

The general target of this policy is that the ratio of days with good air quality (the value of Air Quality Index (AQI) ≤ 100; AQI is a dimensionless index for measuring ambient air quality using six air pollutant concentrations (including PM_2.5_, PM_10_, SO_2_, NO_2_, CO and O_3_; the calculation process of AQI is provided in Appendix B)) in cities will reach 80%. Additionally, specific targets include over 18% reduction in PM_2.5_ concentrations and significant reduction in emissions of major air pollutants (SO_2_ and NO_x_ reduced by more than 15%) between 2015 and 2020. Unlike previous policies that pay more attention to air pollution control at the national level, the BSDW policy focuses more on air pollution control in heavily air polluted regions (including BTH and surrounding areas; Yangtze River Delta; Fenwei plain). Moreover, the BSDW policy emphasizes the pollution management throughout the heating season, which is typically the foremost serious pollution of the year. To achieve these formidable targets, BSDW policy places greater stress on promoting the control of four major sources of air pollution, including industry, coal, diesel trucks and dust, and proposes the following long-term strategies: first, adjust and optimize the industrial structure to achieve green industrial development. BSDW policy attaches special attention to the management of heavy pollution industries. The iron and steel industries, thought to be significant sources of pollution emissions, need to scale back their pollution by adopting measures such as shutdown (132 industrial parks closed in Tianjin [22]), on-site transformation and relocation to promote upgrading (general manufacturing enterprises in Beijing should relocate [23], as well as the Tang Gang, Shi Gang in Hebei province [24]). Second, optimize the energy structure, implement total coal consumption control scheme, and form a clean, low-carbon and efficient energy system. Third, adjust the transportation structure, tighten motor vehicle exhaust emission standards, and establish a cleaner transportation system. Fourth, rationally coordinate the land use structure and improve the control of non-point source pollution. In addition, BSDW policy also ensures effective implementation by improving the monitoring system and strengthening penalties. The policy has been promoted to establish regional co-management projects, such as ultra-low emission transformation of biomass boilers, regional joint environmental monitoring and supervision, which have been found to contribute to the air pollution reduction of BTH region [25,26].

Air pollution exhibits clear regional characteristics [27]. The BTH region is located in the north of the North China Plain, bordering Yanshan Mountain in the north, Bohai Sea in the east and Taihang Mountain in the west. With a total area of 216,000 square kilometers, a population of about 108.605 million and a regional GDP 8.6 trillion yuan, BTH region is amongst one the largest and most dynamic regions in northern China, as well as one of the three most economically developed regions in China [28]. As 12.5% of the world’s iron and steel production capacity is concentrated in Tianjin and Hebei, direct air pollutant emissions from iron and steel industry plants account for 28% of the SO_2_ emissions and 7.5% of the NO_x_ emissions in the BTH region, respectively [29]. In 2019, BTH consumed 9.84% of the total energy consumption of China [30] and contributed to 6.18% of the total SO_2_ emission of China; NO_2_ for 9.95% and particulate matter for 4.37% [23]. The contradiction among high-intensity economic activities, resources and environment has made the BTH region the most serious air pollution region in China [3]. The average concentration of PM_2.5_ in the BTH region was approximately 5.5–7.3 times higher than the safety standards (15 μg/m^3^) of the WHO [31]; more worryingly, the situation would possibly be worse in winter and spring [32].

Many studies have pointed out that the concern and participation of the government (government documents and policies) is the most direct factor affecting environmental quality [33]. Therefore, it is essential to evaluate the impact of these different policies on the environment [34]. Based on the fact that cities should be central subjects of air pollution control, this paper aims to use high-resolution air pollution data to explore whether the effect of the BSDW policy varies in heating seasons, multiple forms of cities and cities in BTH region, so as to provide clear and constructive suggestions for the formulation of air pollution prevention policies in China in the future.

The structure of the article is as follows. In Section 2, we execute a brief literature review; Section 3 is the data description and methodology; Section 4 shows the results and discussion; Section 5 is the conclusion and policy implications.

## 2. Literature Review

Air pollution in the BTH region has attracted the attention of many researchers. Some researchers have performed analyses on the scientific causes of air pollution [35,36] and a number of studies have been conducted on the impact of air pollution [37,38,39,40]. Scholars usually performed econometric models to directly compare the changes of air pollution and test the effects of air pollution control policies, including the difference-in-difference (DID) method [41,42], regression discontinuity design (RDD) [43], propensity score matching (PSM) [44] and the synthetic control method (SCM) [45]. Some recent studies have focused on the impact of the “Action Plan for Air Pollution Prevention and Control” [46,47,48] and the “clean heating” policy [49,50,51]. Huang et al. [52] used hierarchical Bayesian models and daily data to estimate the health impacts of the “Action Plan for Air Pollution Prevention and Control” from 2013 to 2017 in 74 key cities in China, and found that annual average concentrations of PM_2.5_, SO_2_ and CO decreased by 33.3%, 54.1%, 28.2%, while no significant changes were seen in NO_x_ (9.7% reduction; 95% CI −23.0 to 42.4) or O_3_ (20.4% increase; 95% CI −30.1 to 71.0). Guo et al. [53] used the varying coefficient model to analyze the impact of clean electricity utilization on yearly air pollution in the BTH region and identify the long-term relationship between heating and air pollution. The results showed that the coefficient between coal consumption and pollutant emissions was positive, while electricity consumption was negatively correlated with pollutant emissions, which indicated that “replacing coal with electricity” in BTH region had played an active role in air pollution control. There were also analyses showing solicitude for key policy directions of pollution mitigation in the BTH region [54]. Jiang et al. [43] utilized the RDD method and found that the BSDW policy reduced the monthly average concentration of PM_2.5_ and PM_10_ by 14.49 μg/m^3^ and 23.43 μg/m^3^. Xu et al. [55] used the GAINS IV Asia (Greenhouse Gas and Air Pollution Interactions and Synergies) model to assess the potential for yearly air pollution abatement, air quality improvement and associated costs of the BSDW policy in the BTH region.

The above researches provide us with very valuable ideas, and there are still some areas that can be improved. Firstly, few studies have involved a systematic analysis of the impacts of BSDW policy on air quality based on daily scale and monitoring point data [43,55]. Due to the lack of high-resolution geographical data of prefecture-level cities, even those studies based on monthly or daily data failed to identify the policy effect accurately. Therefore, a comprehensive evaluation of the effectiveness of the policy is urgently needed. Secondly, few studies have explored the effect of the BSDW policy in the heating seasons [43], leading to the failure of identifying the policy effect on this special period that had been proven to contribute to serious air pollution. Thirdly, there has been little analysis focusing on different policy effects on different cities [55] and air pollutants. As one of the important topics in the research fields of environmental economics and environmental analyses, it is necessary to assess the pure effect of the implementation of the BSDW policy, which will help to realize the modernization of the government governance system and capabilities of developing countries.

## 3. Data and Methodology

### 3.1. Data

**Figure A1:**
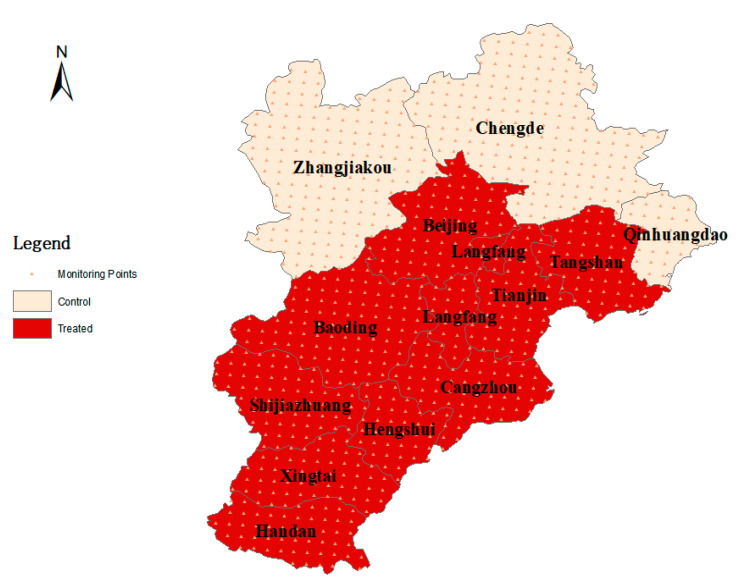
Spatial distribution of sample monitoring points.

Cities in the treatment group and control group are shown in Figure A1, which are in red and pink, respectively. The treatment group contains Beijing, Tianjin, Shijiazhuang, Tangshan, Handan, Xingtai, Baoding, Cangzhou, Langfang and Hengshui, which have begun to take the BSDW policy since 5 July 2018, while the control group includes Zhangjiakou, Chengde and Qinhuangdao, which are not included in the BSDW policy to take pollution control actions. We use China’s high-resolution air pollution reanalysis data [56] from 1 January 2018 to 31 December 2018 to evaluate the impact of the BSDW policy on urban air quality systematically and accurately. The concentrations of pollutants and meteorological data of monitoring points in each city are extracted by ArcMap 10.8 and Python. Figure A1 also depicts all the monitoring points selected in this paper, i.e., 947 monitoring points. The treatment group includes 570 monitoring points. In this paper, the daily average concentrations of various pollutants (PM_2.5_, PM_10_, SO_2_, NO_2_, CO, O_3_) are taken as dependent variables. Moreover, we present the policy effect on the AQI. In order to eliminate the interference of meteorological factors on the results, we specifically control the daily average temperature (temp) [57,58], daily average wind speed (u: longitude wind speed; v: latitude wind speed) [59,60], daily average relative humidity (rh) [61,62] and daily average pressure (psfc) [63] of each monitoring point.

**Figure 1:**
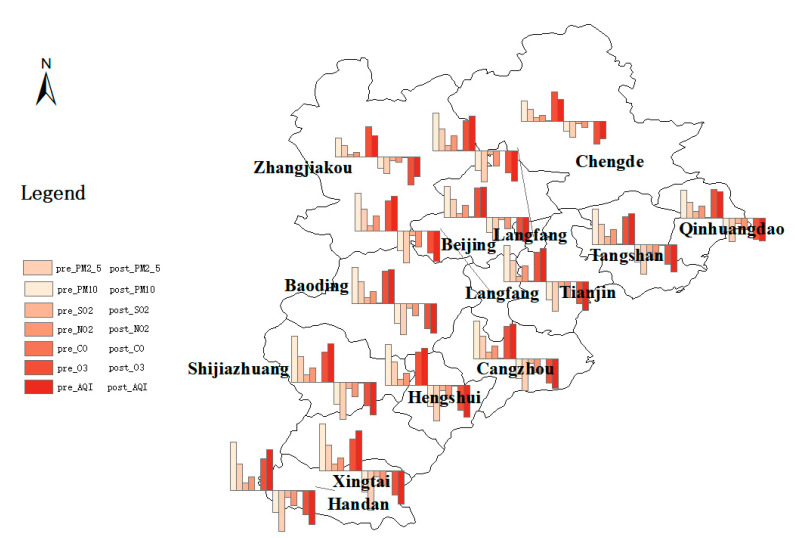
Distribution of air pollutants’ concentration on temporal-spatial scale.

As shown in Table 1 and Table A1, the overall pollution level in BTH region is high, for example, the daily average PM_2.5_ concentration of treatment group is higher than the first level concentration limit standard of GB3095-2012 (35 μg/m^3^) and the standard of WHO’s Air Quality Guidelines (AQG) (15 μg/m^3^). Similarly, the daily average concentration of PM_10_ is also significantly higher than 50 μg/m^3^ (the first level concentration limit standard of GB3095-2012) and 45 μg/m^3^ (WHO (AQG 2021)). The daily average concentrations of SO_2_, CO and O_3_ are lower than WHO (AQG 2021) standard (40 μg/m^3^; 4 mg/m^3^; 100 μg/m^3^) and first level concentration limit standard of GB3095-2012 (50 μg/m^3^; 4 mg/m^3^; 100 μg/m^3^). For NO_2_, the daily average concentration of the treatment group is higher than WHO (AQG 2021) standard (25 μg/m^3^) but lower than first level concentration limit standard of GB3095-2012 (80 μg/m^3^). The maximum and minimum values of all air pollutants concentrations imply that the BTH region’s air pollution has obvious regional differences. Figure 1 (derived from Table 1) shows the concentration distribution of air pollutants in the BTH region before (from 1 January 2018 to 4 July 2018; the air pollution concentration histogram is located above the horizontal axis) and after (from 5 July 2018 to 31 December 2018; the air pollution concentration histogram is located below the horizontal axis) the implementation of the BSDW policy. From Table 1 and Figure 1, we can draw a preliminary conclusion that the pollutant concentration in the treatment group decreased more than that in the control group after the implementation of the policy.

### 3.2. Empirical Model

To assess the impact of China’s BSDW policy on the local air quality, this paper utilizes the DID method, which is widely used in the estimation of the causal effect of a treatment by using longitudinal data from the treatment and control group [64,65,66]. The DID model can control the systematic differences between the treatment and control groups and isolate the changes in the outcomes over time between the samples that were and were not affected by the policy [67]. It can also remove the biases that could be the result of trends caused by other factors (i.e., missing variables). The DID model can be defined as follows:

(1) $$Air\;quality_{i,t}=\beta_{0}+\beta_{1}treat_{i,t}+\beta_{2}time_{i,t}+\beta_{3}time_{i,t}\times treat_{i,t}+\beta_{4}X_{i,t}+\alpha_{i}+\delta_{t}+\varepsilon_{i,t}$$

(2) $$\mathrm{treat}=\left\{ \begin{array}{l} 0,\;\;\mathrm{control}\;\mathrm{group}\\ 1,\;\;\mathrm{treatment}\;\mathrm{group} \end{array}\right.$$

(3) $$\mathrm{time}=\left\{ \begin{array}{l} 0,\;\mathrm{before}\;\mathrm{policy}\;\mathrm{implementation}\\ 1,\;\mathrm{after}\;\mathrm{policy}\;\mathrm{implementation} \end{array}\right.$$

where the dependent variable *Air quality_i,t_* refers to the monitoring station’s daily concentration of PM_2.5_, PM_10_, SO_2_, NO_2_, CO, O_3_ and other air pollutant used in the robustness analysis (AQI). *treat_i,t_* represents the monitoring station *i* at day *t*. It is equal to 1 if the monitoring station is located in the city where the policy is implemented, otherwise it is equal to 0. *time_i,t_* is a dummy variable that represents the BSDW policy and is equal to 1 since 5 July 2018, otherwise it is equal to 0. *time_i,t_ × treat_i,t_* notes the interaction item of these two dummy variables. *X_i__,__t_* represents control variables related to the environmental pollution, namely the meteorological factors (including daily temperature, daily longitude wind speed, daily latitude wind speed, relative humidity and daily surface pressure). The individual-fixed effects *α_i_* and the day-fixed effects *δ_t_* are included to exclude the interference of other possible confounders. *β_i_* is the vector of the coefficients of each independent variable. *β*_0_ represents the constant term. *β*_1_ and *β*_2_ represent the coefficient of *treat_i,t_* and *time_i,t_*, respectively. The coefficient of *time_i,t_ × treat_i,t_* (*β*_3_) captures the reduction effect of the policy on the treatment group [68], namely the concentration reduction amount of air pollutants, which can effectively avoid the endogenous problems caused by omitted variable bias and accurately reveal the net policy impact [69]. The basic principle of DID method is to build the difference-in-difference statistics reflecting the policy effect, that is, the coefficient of the interaction term *β*_3_, by comparing the differences between the control group and the treatment group before and after the implementation of the policy, so as to obtain the desired net effect of the policy [70]. A negative estimated coefficient (statistically significant) denotes a significant reduction in the pollutant concentration in the region because of the BSDW policy, indicating that the emission-control policy has played a positive and significant role. *ε_i__,__t_* denotes random disturbance term.

## 4. Results and Discussion

### 4.1. Unit Root Tests and Hausman Tests

We proceeded with the unit root diagnostic test; namely, the Levin, Lin and Chu test was used to examine the stationary processes of our variables [71]. The results suggested that all variables were stationary at level. Then, we applied Hausman test to test whether individual effects were independent from other explanatory variables, so as to determine the applicability of the fixed effect model and random effect model [72]. The original hypothesis of Hausman test is that individual effects are independent from other explanatory variables, namely the random effect model, and the results significantly reject the original hypothesis, which indicates that the fixed effect model based on within ordinary least square (OLS) estimation method is better than random effect model based on the feasible generalized least square (FGLS) estimation method. Furthermore, the modified Hausman statistic is used for a more accurate test, and the results also confirm the applicability of the fixed effect model. After testing, the panel had sequence correlation and heteroscedasticity, so the classical Hausman test was no longer applicable. Therefore, to address the heteroscedasticity problem, Hausman based on bootstrap method was used for further tests. The results further indicate that the fixed effect model should be chosen to obtain consistent estimation results.

### 4.2. Empirical Results

The results of the DID regression analysis are presented in Table 2. Columns (1), (3), (5), (7), (9) and (11) represent the results where we take the effect of meteorological factors on concentrations of air pollutants into consideration, that is, the meteorological factors are included in our empirical model as control variables, so as to obtain a more precise estimation of policy effect, while the other columns do not. Columns (1) and (2) show that the BSDW policy has significantly reduced the urban PM_2.5_ concentration after its release. As shown in Column (1), the estimated coefficient of policy variable is −4.639 under 1% significance. This result is similar but larger than that of Jiang et al. [43], who found that PM_2.5_ was reduced by 0.2637 μg/m^3^ after the policy taking place. Column (2) represents that the estimated coefficient of policy variable is −1.951 under 1% significance. Obviously, there is no change in the direction and significance of the coefficient of policy variable. Wang et al. [73] showed that the main sources of PM_2.5_ in BTH region were secondary nitrate (36–58%), traffic (8–26%), residential coal combustion (8–16%) and biomass burning (5–16%). Among them, secondary nitrate is formed by photochemical transformation of NO_2_ with significantly high photochemical activity [74]. In line with the main sources of PM_2.5_, BSDW policy attaches great importance to implementing total coal consumption control and actively adjusting transportation structure and developing green transportation system. Therefore, the underlying reason for the emission reduction effect of BSDW policy lies in that it inhibits the production of pollutants from the source. Similarly, from the perspective of PM_10_ (columns (3), (4)), SO_2_ (columns (5), (6)), CO (columns (9), (10)) and O_3_ (columns (11), (12)), the results also signify that the BSDW policy has significantly reduced the pollution in cities. As presented in column (10), the estimated coefficient of the policy variable is −0.057 under 1% significance. Column (9) controls the meteorological factors, and the results show that the corresponding coefficient is −0.014 under 1% significance. Similarly, as for O_3_, the estimated coefficient of the policy variable is −7.882 under 1% significance, which is shown in column (12). After controlling meteorological factors, the results are basically consistent.

However, the concentration of NO_2_ increased significantly when the BSDW policy came into force. As shown in column (7), the estimated coefficient of the policy variable after controlling the meteorological factors is 1.865 under 1% significance. Evidence exists that NO_2_ has negative environmental-human health consequences, especially in circumstances where prolonged exposure is experienced [75]. Nitrogen oxides are mainly produced by anthropogenic activities, including vehicular emissions, biomass burning, fossil fuel combustion, thermal power plants, domestic solid fuel use, while natural sources include lightning and soils [76,77,78]. Matandirotya and Burger [79] found that seasonal biomass burning, which increased during the winter season, was also related to the emission of nitrogen oxides. However, the BSDW policy incentives the usage, promotion, and transformation of biomass power generation; specifically, it proposes to encourage the development of county biomass cogeneration, biomass briquette boilers and biological natural gas where resources are available. In view of the fact that the policy did not refine the specific indicators, the proposal to implement ultra-low emission transformation of biomass boilers may not reduce the emission of nitrogen oxides. Moreover, the efforts to reduce the emission of nitrogen oxides caused by domestic solid fuel use and thermal power plants may fail due to the lack of explicit emission reduction targets, although existing technologies such as selective catalytic reduction of NO_x_ with NH_3_ can make NO_x_ emissions meet the standard [80].

The results of the DID regression analysis on AQI and different regions are presented in Table A2 and Table A3. Based on the results, the BSDW has significantly reduced the urban AQI concentration and air pollutants in Beijing, Tianjin, and Hebei province, which shows that the results in this paper are robust.

### 4.3. Robustness Test

#### 4.3.1. Parallel Trend

**Figure 2:**
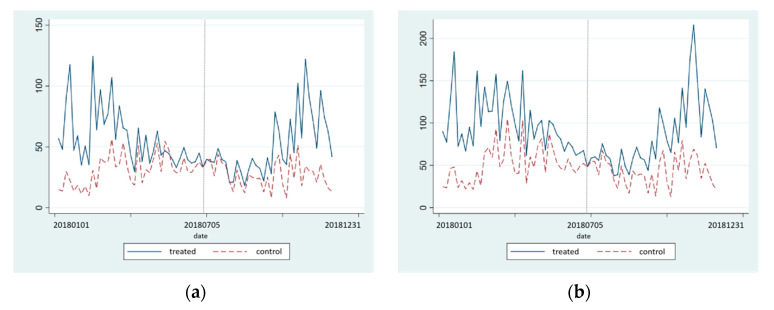

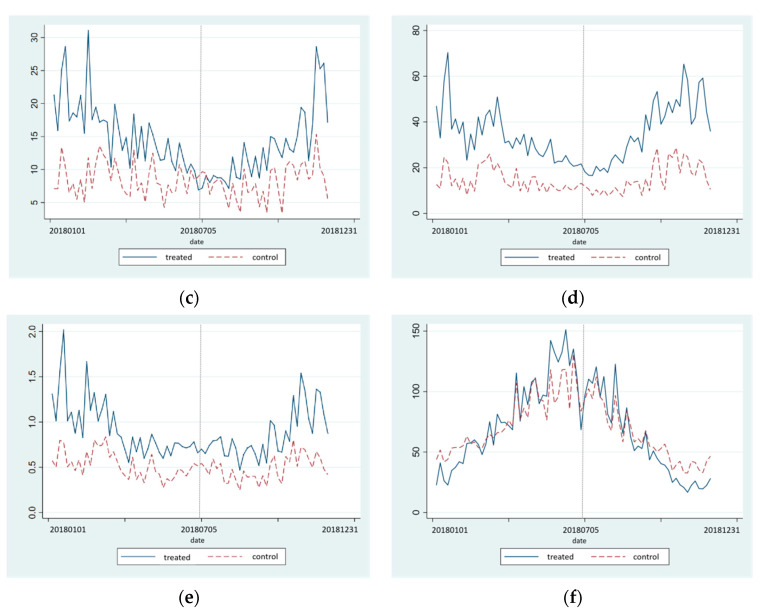
Parallel trend test ((**a**) PM_2.5_, (**b**) PM_10_, (**c**) SO_2_, (**d**) NO_2_, (**e**) CO, (**f**) O_3_).

We investigate whether the DID parallel trend is likely to exist. The original hypothesis of the parallel trend test is that before the implementation of the policy, the change trend of target variables over time is basically the same in the treatment group and the control group. To assess the plausibility of the parallel trend, the mean of the dependant variables for the treatment group and the control group can be plotted to assess the plausibility of the parallel trend [66,81]. Figure 2. indicates that the different groups appear to evolve along a similar temporal path, at least prior to treatment. After the implementation of the BSDW policy in the treatment group, a significant difference in slope can be detected after the implementation of the BSDW policy (in Figure 2 a,c,d,f). Thus, existing evidence indicates that parallel trends roughly exist, therefore, it can be concluded that the results based on DID method in Section 4.2 are robust.

#### 4.3.2. Effect of Unobserved Variables

In addition to the parallel trend assumption test, we also test the effect of unobserved variables. The DID method is helpful for reducing the number of control factors to be considered in the model. To eliminate the effect of unobserved variables, this paper controlled the regional characteristics and adopted the two-way fixed effect model in the basic regression. However, it may still be difficult to observe and control some factors that change over time and space. Specifically, different industrial policies carried out by local governments in different regions may affect the upgrading of manufacturing industry, finally resulting in the estimation error. In order to eliminate the effect of unobserved factors, following the studies of La Ferrara et al. [82] and Liu and Lu [83], this paper uses the following method to indirectly test whether the unobserved characteristics of these regions will affect the estimation results.

According to the estimation in Equation (1), the formula for calculating the estimated coefficient of *time_i,t_ × treat_i,t_* is as follows:

(4) $$\overset{\wedge }{\beta_{3}}=\beta_{3}+\gamma \frac{\mathrm{cov}(\;time_{i,t}\times treat_{i,t},\varepsilon_{i,t}|X_{i,t})}{\mathrm{var}(\;time_{i,t}\times treat_{i,t}|X_{{}_{i,t}})}$$

**Figure 3:**
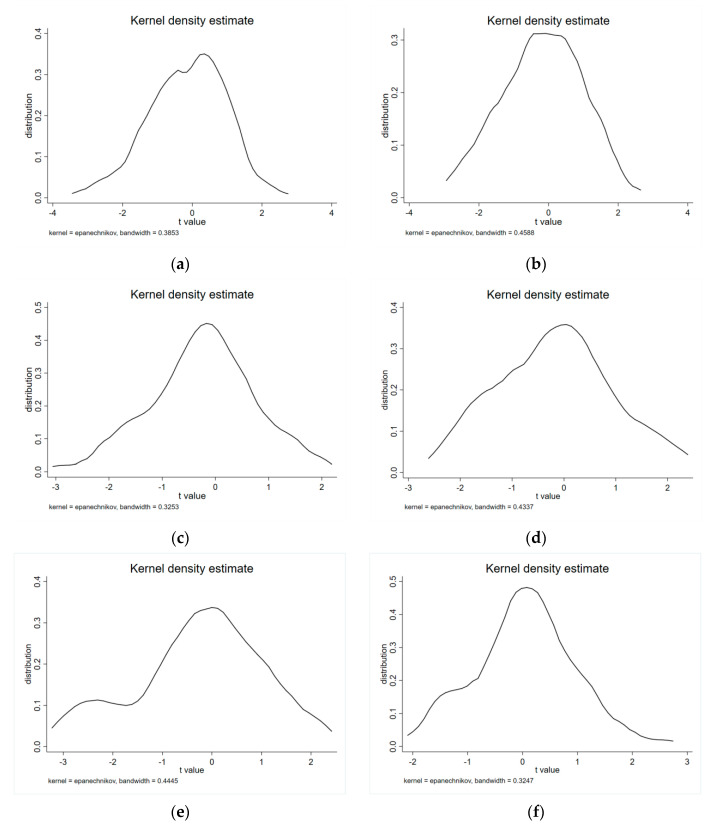
Distribution of estimated coefficients ((**a**) PM_2.5_, (**b**) PM_10_, (**c**) SO_2_, (**d**) NO_2_, (**e**) CO, (**f**) O_3_).

In Equation (4), *time_i,t_ × treat_i,t_* notes the interaction item of two dummy variables (*treat_i,t_* and *time_i,t_*). *β*_3_ (the coefficient of *time_i,t_ × treat_i,t_*) reflects the net effect of the policy. *X_i,t_* represents all the control variables included in this paper. *i* and *t* represent the monitoring station *i* and day *t*, respectively. *ε_i__,__t_* denotes random disturbance term. Theoretically, if *γ* = 0, it means that the unobserved factors will not interfere with the estimated results and thus *β_3_* is unbiased. However, it is difficult to directly test whether *γ* is 0. Therefore, if we replace the *time_i,t_ × treat_i,t_* with a variable, which will not have real effect on the *Air quality_i,t_* in theory, and its coefficient is estimated to be zero, then *γ* = 0 can be derived inversely from this result. Thus, this paper randomizes the impact of the BSDW policy on specific regions (generated by computer), and then repeats this exercise 100 times. Such random processing can ensure that the BSDW policy will not affect the *Air quality_i,t_*, namely *γ* = 0. In this case, the mean value of *γ* is estimated, and the distribution of *γ* is shown in Figure 3. The average value of *γ* is close to zero and not significant compared with the basic results. It can be further found from Figure 3. that *γ* obtained from 100 random processes is distributed around 0, so we can deduce from the inverse that *γ* = 0. These results further boost our confidence that our findings are not severely biased by the unobserved time-variant regional characteristics.

### 4.4. Heterogeneity Analysis

#### 4.4.1. Different Industry Type of Cities

Regional development difference is one of the prominent issues in China’s sustainable and coordinated development [84]. Differences exist in the resource endowment and development path of different regions, leading to the industrial structures various in different regions [53]. Because the industrial structure has a decisive impact on resource consumption and pollutant emissions [85,86] and the content of the BSDW policy implemented in all cities is the same, we further classify cities according to industry types to estimate the emission reduction effect of the BSDW policy. Following the research of Yu et al. [44], this paper divides the treated cities in BTH region into two groups: one is urban consumption city (including Baoding, Beijing, Tianjin, Langfang, Qinhuangdao and Shijiazhuang); the other is resource intensive city (including Xingtai, Handan, Chengde, Zhangjiakou, Tangshan, Cangzhou and Hengshui). In accordance with the energy utilization characteristics of BTH region, including energy intensity and per capita consumption of implied energy, the cities in BTH region can be divided into urban consumption cities and resource intensive cities. Specifically, the urban consumption cities refer to the developed metropolis dominated by service industry and high-end manufacturing, with rapid economic development, low energy intensity but high implied energy per capita consumption. Resource intensive cities rely on local natural resources for development, and thus are energy intensive, with high energy intensity but low implied energy per capita consumption. They often rely on abundant energy to develop high-energy consumption sectors, such as mining sector. The proportion of industry is relatively high. The regression results are shown in Table 3.

In urban consumption cities, rows (1)–(6) show that the emission reduction effects of the BSDW policy on PM_2.5_, PM_10_, SO_2_, NO_2_, and O_3_ are −1.751, −4.503, −1.634, 0.943 and −6.950 under 1% significance. However, the emission reduction effect of CO is no longer significant. In resource intensive cities, the results show that the emission reduction effects of the BSDW policy variable are −2.579, −3.487, −2.162, 3.021, −0.033 and −8.389, respectively. These results are consistent with the results in Section 4.2. However, it is obvious that the policy effect varies from region to region. In resource intensive cities, the BSDW policy can better play its emission reduction effect on PM_2.5_, SO_2_, CO and O_3_.

There are many energy-production and processing industries in resource intensive areas, such as mining and metallurgy [44]. Therefore, resource intensive cities have great potential for energy conservation, and should be the key area for energy conservation and emission reduction. Although there are abundant energy resources in the local area, the energy exploited has been encouraged to be used locally, processed into high-energy consumption products, and exported. Additionally, the energy cost and access threshold of high-energy consumption sectors are very low, and even encouraged. All of the above realities lead to the fact that resource intensive cities do not play the potential of energy conservation. On the one hand, the demand for high-energy consumption areas continues to grow. On the other hand, expanding the capacity of high-energy consumption sectors is the most convenient way for energy-oriented regions to promote economic growth, which drives the transfer of high-energy consumption sectors between the two types of regions, as well as the transfer of energy conservation and emission reduction pressure. In other words, the demand for high-energy consumption products in other regions is the reason for the pressure of energy conservation and emission reduction in resource intensive cities. The BSDW policy emphasizes that cities should speed up the adjustment of energy structure to build a clean, low-carbon and efficient energy system. Thus, resource intensive cities have great potential for energy transformation.

#### 4.4.2. Key Environmental Protection Cities and Other Cities

Population and capital are most concentrated in cities, so is the environmental pressure. Speeding up the process of urbanization is an important way for China to achieve the goal of building a moderately prosperous society in an all-round way. China’s urbanization is in the stage of accelerated development, urban environmental protection work will directly affect the success of national environmental protection work. On 26 November 2007, the State Council issued the “Eleventh Five Year Plan for national environmental protection” [87], which clearly stipulated 113 key urban environmental protection cities. The comprehensive prevention and control of air pollution in 113 key environmental protection cities such as Beijing and Tianjin are the focus of China’s air pollution prevention, as well as the control and efforts to improve the quality of urban and regional air environment. The population of these 113 key environmental protection cities accounts for 50.1% of China’s urban population, and the GDP accounts for 71.3% [43]. At the same time, the plan also clearly proposed to focus on 113 cities to comprehensively promote urban environmental protection work. Therefore, according to the plan, the treated cities in BTH region can be divided into two groups, one is the key environmental protection city, the other is not the key environmental protection city. The regression results are shown in Table 4.

**Figure 4:**
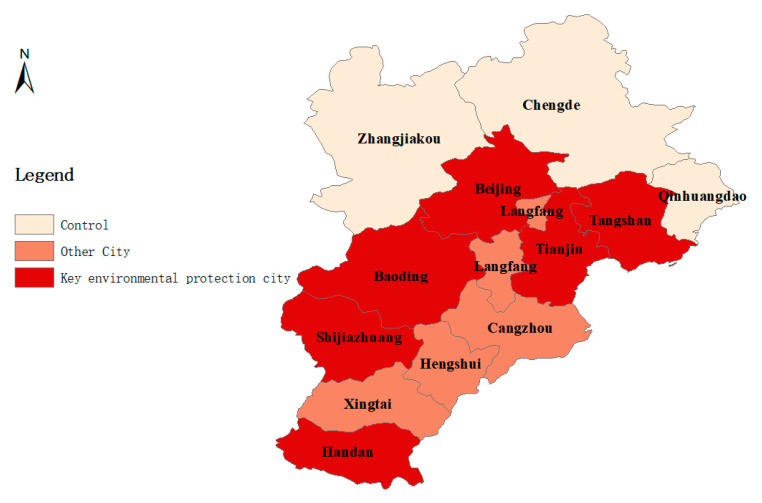
Distribution of key environmental protection cities and other cities.

Rows (1)–(12) can further verify the robustness of the basic regression results. Whether it is a key environmental protection city or not, the BSDW policy will significantly reduce the concentration of air pollutants, except for NO_2_. For PM_10_, the estimated coefficient of the policy variable in key environmental protection cities is −4.323 under 1% significance, and the corresponding coefficient in other cities is −2.396 under 1% significance. As shown in Table 4, compared with the key environmental protection cities, the emission reduction effect of the BSDW policy in other cities is generally low. Figure 4 depicts the distribution of key environmental protection cities and other cities. The industrial distribution in BTH region plays an important role. Heavy industry leads to massive energy consumption (most of which is still provided by coal), and the most polluting is in the south of BTH region, which leads to serious air pollution [88].

According to the results in Table 3, the estimated coefficient of the BSDW policy in key environmental protection cities are always lower than those of other cities, except for SO_2_, CO and O_3_. This shows that after the implementation of the BSDW policy, compared with other cities, the emission reduction effect on PM_2.5_, PM_10_ and NO_2_ in key environmental protection cities is better. Therefore, we can consider including more cities in the list of key environmental protection cities to further improve the air quality in other cities. For SO_2_, however, some studies have pointed out that the combustion of coal in boilers is associated with the release of air pollutants, especially SO_2_ and total suspended particles (TSP) [89]. The BSDW policy puts forward that “cities should further eliminate the combustion of coal in boilers”. By comparing the government work reports of all cities in 2019, it can be found that almost all key environmental protection cities (such as Beijing, Tianjin, etc.) do not clearly mention the index of coal-fired boiler transformation, while most other cities (such as Xingtai, Zhangjiakou, etc.) have explicitly mentioned the results of coal-fired boiler transformation. For example, Xingtai pointed out that 59 coal-fired boilers with 35 steam tons and below, 671 low nitrogen combustion boilers were eliminated [90]. Besides, 1663 coal-fired boilers were eliminated in Zhangjiakou [91] and “2695 coal-fired boilers were eliminated and banned” in Chengde [92] in the government word reports. Thus, the SO_2_ emission reduction effect in other cities is better than key environmental protection cities.

#### 4.4.3. Heating Seasons

Existing literature [93,94,95] identified that heating in winter increased regional air pollution, which has become the focus of the BSDW policy [88]. Therefore, this paper further identifies the effect of the BSDW policy in heating seasons. According to the planning for heating seasons of the government, the heating season of the BTH region in this paper consists of two periods: the first period is from 1 January 2018 to 14 March 2018, and the second period is from 15 November 2018 to 31 December 2018. The results are displayed in Table 5.

The results in columns (3), (5) and (6) are consistent with the basic regression results, that imply, after controlling the meteorological factors, the BSDW policy will significantly reduce the concentration of SO_2_, CO and O_3_ in heating seasons in the BTH region. For SO_2_, the estimated coefficient of the policy variable is −1.316 under 1% significance. As for CO and O_3_, a significant decrease also exits, with the estimated coefficients of the policy variable being −0.023 and −8.558 under 1% significance, respectively. The most striking observation to emerge from the result is, as shown in columns (1), (2) and (4), this paper finds that the implementation of the BSDW policy does not reduce the concentration of PM_2.5_, PM_10_ and NO_2_; on the contrary, it causes a significant increase in the concentration of these air pollutants. For PM_2.5_, PM_10_ and NO_2_, the estimated coefficients of the policy variable are 2.099, 15.126 and 5.527 under 1% significance, respectively. This is a rather disappointing result, which indicates that with the implementation of the BSDW policy, the concentrations of PM_2.5_, PM_10_ and NO_2_ increase significantly. However, this result has not previously been described. A possible explanation for these results may be since the lack of adequate natural gas supply sources, the original coal-fired heating facilities were not demolished, which induces that it is difficult to promote the “continue to promote coal to electricity and coal to gas” proposed by the BSDW policy.

#### 4.4.4. City Heterogeneity

Since 2013, the emergency response plan of heavy air pollution in the BTH region has gradually tended to a unified emergency early warning response standard. Under different levels of heavy pollution weather warning, different cities and municipalities should formulate and implement cost-effective and differentiated heavy pollution emergency plans according to their actual situations, namely “one city, one policy” [96]. Therefore, this paper explores the different influences of the BSDW policy on different cities. The estimation results of the BSDW policy on different cities are shown in Table 6.

**Figure 5:**
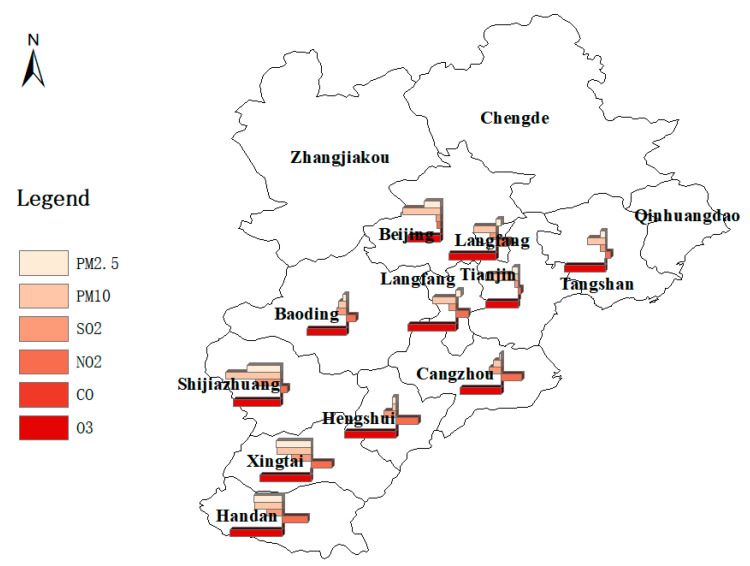
Estimation results of the BSDW policy on different cities.

As shown in Figure 5, for PM_2.5_, the cities with the best pollutant reduction effect are Xingtai, Shijiazhuang and Handan; for PM_10_, Shijiazhuang, Beijing and Xingtai are the cities with the largest decrease in pollutant concentration; for SO_2_, emission reduction effect in Shijiazhuang, Xingtai and Handan are the best; for NO_2_, only Beijing has achieved significant emission reduction of NO_2_; the concentration of CO decreases significantly in Hengshui, Cangzhou, Tangshan, Xingtai and Shijiazhuang; for O_3_, the pollutant emission reduction effect in Handan, Hengshui and Xingtai are the best. On the whole, the emission reduction effect of cities located in the south of the BTH region are better and the cities in the north of the BTH region have great potential for pollutant emission reduction. Based on the above results, the local governments of different cities should formulate specific measures to implement the BSDW policy according to their own characteristics.

## 5. Conclusions and Policy Implications

There is an urgent need to improve air quality in China’s BTH region. The three-year Action Plan to Win the BSDW has been implemented on a large scale. This paper has taken the implementation of the BSDW policy in the BTH region as a quasi-natural experiment and regarded the BSDW policy as an exogenous policy impact. Using daily panel data from 947 grid monitoring points in the BTH region in 2018, this paper sets out to analyze the emission reduction effect of China’s air governance policies in BTH region by applying DID model. Furthermore, focusing on various pollutants, different city types, heating seasons and city level, this paper explores the different effects of the BSDW policy. The main conclusions are as follows.

Our findings propose that the BSDW policy has partly significantly improved overall air quality and reduced atmospheric pollutant emissions in the BTH region. The policy leads to a reduction in the daily concentration of PM_2.5_, PM_10_, SO_2_, CO and O_3_ by −4.639, −8.84, −2.657, −0.057 and −7.882 under 1% significance, respectively. After controlling the meteorological factors, the estimated coefficient of the policy variable increases to −1.951 (PM_2.5_), −3.872 (PM_10_), −1.902 (SO_2_), −0.014 (CO) and −7.882 (O_3_) under 1% significance. The parallel trend test results and the test of effect of unobserved variables indicate that the reduction in air pollution concentration after 4 July 2018 is due to the BSDW policy, rather than other factors. Substituting the dependent variable with AQI and dividing the treatment group into three groups obtained consistent results; the concentration of AQI (−3.012) has decreased significantly after controlling the meteorological factors, indicating that the conclusions in this paper are robust. The heterogeneity of different city types and different cities also shows that the aforementioned conclusions are robust. On the whole, the emission reduction effect on cities located in the south of the BTH region is better. Resource intensive cities, non-key environmental protection cities and cities in the north of the BTH region have great potential for pollutant emission reduction. The results of the heterogeneity analysis show that the estimated coefficients of the policy variable in key environmental protection cities are always lower than those of other cities, except for SO_2_, CO and O_3_.

Despite these promising results, questions remain. One of the more significant findings to emerge from this study is that with the implementation of the BSDW policy, the concentration of NO_2_ increases significantly, with the estimated coefficient of 0.673 under 1% significance. The corresponding coefficient is 1.865 under 1% significance after controlling the meteorological factors. Another important finding is that in heating seasons, the BSDW policy will reduce the concentration of SO_2_ (−1.316), CO (−0.023) and O_3_ (−8.558) under 1% significance. Surprisingly, the concentrations of PM_2.5_ (2.099), PM_10_ (15.126) and NO_2_ (5.527) increase significantly in heating seasons, which might be of interest to policymaking.

According to the findings, several suggestions have been proposed to further promote the implementation of the BSDW policy. First, set and aim to achieve a specific goal based on city level to expedite an optimal “Blue Sky” strategy. Different cities should formulate and implement differentiated air pollution prevention plans according to local actual situations, namely “one city, one policy”. In addition, incorporating more cities into the list of key environmental protection cities may contribute to the improvement of the air quality in other cities. Second, refine the specific emission reduction indicators related to domestic solid fuel use and thermal power plants. Besides, attention should be paid to accelerate the transformation of gas-fired boilers with low nitrogen and urban biomass boilers with ultra-low emissions and formulate specific transformation indicators. Third, further improve the natural gas supply system in the BTH region, and clean heating diffusion should be encouraged and enforced.

## Figures and Tables

**Table 1:** Descriptive statistics.

	Variable	Variable Description	Group	Pre_Policy					Post_Policy					Difference
				Obs	Mean	Std.Dev.	Min	Max	Obs	Mean	Std.Dev.	Min	Max	
Dependant variable	PM_2.5_	Daily PM_2.5_ concentration (μg/m^3^)	treatment group	105,450	57.819	40.916	2.104	401.410	102,600	49.441	37.332	1.229	401.094	−8.378
			control group	69,745	31.030	23.472	0.787	248.346	67,860	27.291	21.417	0.811	273.353	−3.739
	PM_10_	Daily PM_10_ concentration (μg/m^3^)	treatment group	105,450	100.332	57.993	3.158	533.643	102,600	82.881	57.701	1.805	558.074	−17.451
			control group	69,745	51.732	38.792	1.175	434.506	67,860	43.122	32.389	1.086	332.930	−8.610
	SO_2_	Daily SO_2_ concentration (μg/m^3^)	treatment group	105,450	16.063	9.880	0.561	98.555	102,600	12.882	9.093	0.510	181.762	−3.181
			control group	69,745	8.624	6.440	0.458	74.541	67,860	8.100	6.162	0.331	84.790	−0.524
	NO_2_	Daily NO_2_ concentration (μg/m^3^)	treatment group	105,450	34.146	17.744	1.202	138.518	102,600	35.388	19.303	1.071	126.222	1.242
			control group	69,745	14.575	12.561	0.524	115.178	67,860	15.144	13.356	0.388	88.399	0.569
	CO	Daily CO concentration (mg/m^3^)	treatment group	105,450	0.944	0.538	0.158	6.218	102,600	0.846	0.451	0.147	4.374	−0.098
			control group	69,745	0.527	0.353	0.135	5.392	67,860	0.487	0.311	0.138	3.798	−0.040
	O_3_	Daily O_3_ concentration (μg/m^3^)	treatment group	105,450	79.198	40.298	4.407	216.773	102,600	59.094	37.708	0.365	210.037	−20.104
			control group	69,745	75.785	30.060	9.867	200.445	67,860	63.563	28.236	0.866	196.362	−12.222
	AQI	Daily Air Index	treatment group	105,450	90.014	46.585	18.344	434.273	102,600	75.993	44.113	16.601	458.074	−14.021
			control group	69,745	56.363	27.583	18.679	318.133	67,860	48.402	24.165	15.861	323.353	−7.961
Control variable	temp	Daily temperature (K)	treatment group	105,450	285.135	12.526	254.256	308.081	102,600	288.772	11.290	254.852	308.564	3.637
			control group	69,745	277.893	13.374	245.016	304.274	67,860	281.764	12.228	246.046	303.788	3.871
	u	Daily longitude wind speed (m/s)	treatment group	105,450	−0.006	2.983	−13.084	15.174	102,600	0.064	2.501	−11.774	14.382	0.070
			control group	69,745	2.351	3.543	−11.984	17.524	67,860	2.012	3.250	−10.483	16.534	−0.339
	v	Daily latitude wind speed (m/s)	treatment group	105,450	−0.014	4.085	−16.738	12.715	102,600	−0.714	3.409	−15.413	10.081	−0.700
			control group	69,745	−0.878	4.332	−17.114	12.920	67,860	−0.749	4.058	−16.205	11.034	0.129
	rh	Relative humidity (%)	treatment group	105,450	39.578	17.010	7.438	100.000	102,600	47.790	19.985	6.676	99.512	8.212
			control group	69,745	46.691	16.234	7.968	100.000	67,860	57.711	18.352	16.399	100.000	11.020
	psfc	Daily surface pressure (Pa)	treatment group	105,450	100,237.440	2720.619	86,822.117	104,083.490	102,600	100,324.770	2684.693	87,136.297	104,536.200	87.330
			control group	69,745	90,485.796	4712.600	81,700.281	104,072.480	67,860	90,724.038	4640.664	82,274.836	104,507.820	238.242

**Table 2:** Basic effects of BSDW policy on pollution.

Variables	PM_2.5_		PM_10_		SO_2_		NO_2_		CO		O_3_	
	(1)	(2)	(3)	(4)	(5)	(6)	(7)	(8)	(9)	(10)	(11)	(12)
time	−11.743 ***	−6.651 ***	−20.527 ***	−14.003 ***	−4.801 ***	−2.759 ***	−12.958 ***	−2.782 ***	−0.349 ***	−0.181 ***	7.606 ***	3.829 ***
	[1.023]	[1.157]	[1.570]	[1.696]	[0.280]	[0.203]	[0.547]	[0.459]	[0.017]	[0.014]	[0.705]	[0.340]
treat	18.217 ***	16.995 ***	22.445***	23.773 ***	−2.091 ***	−1.108 **	2.501	1.030	−0.033	−0.078 ***	12.055 ***	13.069 **
	[1.466]	[1.093]	[3.443]	[2.201]	[0.673]	[0.521]	[1.718]	[1.204]	[0.038]	[0.023]	[1.831]	[0.488]
time × treat (*β*_3_)	−1.951 ***	−4.639 ***	−3.872 ***	−8.84 ***	−1.902 ***	−2.657 ***	1.865 ***	0.673 ***	−0.014 ***	−0.057 ***	−7.882 ***	−7.882 ***
	[0.160]	[0.178]	[0.313]	[0.348]	[0.097]	[0.109]	[0.093]	[0.087]	[0.003]	[0.003]	[0.351]	[0.348]
Controls	YES	NO	YES	NO	YES	NO	YES	NO	YES	NO	YES	NO
Time_FE	YES	YES	YES	YES	YES	YES	YES	YES	YES	YES	YES	YES
City_FE	YES	YES	YES	YES	YES	YES	YES	YES	YES	YES	YES	YES
Constant	−385.773 ***	−50.208 ***	−1126.596 ***	82.962 ***	−201.496 ***	24.384 ***	−332.485 ***	47.924 ***	−8.114 ***	1.400 ***	323.930 ***	16.614 ***
	[28.835]	[1.158]	[51.439]	[1.757]	[8.462]	[0.527]	[17.551]	[1.001]	[0.438]	[0.020]	[21.657]	[0.575]
N	345,655	345,655	345,655	345,655	345,655	345,655	345,655	345,655	345,655	345,655	345,655	345,655
within R2	0.592	0.565	0.638	0.605	0.568	0.524	0.678	0.645	0.582	0.549	0.856	0.842

Notes: Robust standard errors are clustered at city level in parentheses; **, *** represent 5%, and 1% significance levels, respectively.

**Table 3:** Estimation results of the BSDW policy on air pollution in different industry type of cities.

Region		Variables	Time × Treat (*β*_3_)	Constant	Controls	Time_FE	City_FE	within R^2^	N
urban consumption cities	(1)	PM_2.5_	−1.751 ***	−30.648 ***	YES	YES	YES	0.624	248,200
			[0.212]	[31.226]					
	(2)	PM_10_	−4.503 ***	−585.202 ***	YES	YES	YES	0.671	248,200
			[0.383]	[45.925]					
	(3)	SO_2_	−1.634 ***	−141.805 ***	YES	YES	YES	0.591	248,200
			[0.119]	[8.102]					
	(4)	NO_2_	0.943 ***	−600.941 ***	YES	YES	YES	0.694	248,200
			[0.113]	[20.921]					
	(5)	CO	0.005	−4.899 ***	YES	YES	YES	0.607	248,200
			[0.004]	[0.487]					
	(6)	O_3_	−6.950 ***	248.841 ***	YES	YES	YES	0.852	248,200
			[0.378]	[19.720]					
resource intensive cities	(7)	PM_2.5_	−2.579 **	−600.941 ***	YES	YES	YES	0.539	235,060
			[0.207]	[27.957]					
	(8)	PM_10_	−3.487 ***	−1421.910 ***	YES	YES	YES	0.600	235,060
			[0.355]	[59.018]					
	(9)	SO_2_	−2.162 ***	−247.126 ***	YES	YES	YES	0.543	235,060
			[0.119]	[10.447]					
	(10)	NO_2_	3.021 ***	−457.140 ***	YES	YES	YES	0.636	235,060
			[0.124]	[19.885]					
	(11)	CO	−0.033 ***	−12.514 ***	YES	YES	YES	0.541	235,060
			[0.003]	[10.519]					
	(12)	O_3_	−8.389 ***	405.019 ***	YES	YES	YES	0.834	235,060
			[0.334]	[22.845]					

Notes: Robust standard errors are clustered at city level in parentheses; **, *** represent 5%, and 1% significance levels, respectively.

**Table 4:** Estimation results of the BSDW policy on air pollution in the environmental protection cities and other cities.

Region		Variables	Time × Treat (*β*_3_)	Constant	Controls	Time_FE	City_FE	within R^2^	N
Key environmental protection cities	(1)	PM_2.5_	−1.987 ***	−337.335 ***	YES	YES	YES	0.586	280,685
			[0.181]	[36.260]					
	(2)	PM_10_	−4.323 ***	−1006.461 ***	YES	YES	YES	0.636	280,685
			[0.352]	[58.666]					
	(3)	SO_2_	−1.661 ***	−210.245 ***	YES	YES	YES	0.540	280,685
			[0.115]	[8.987]					
	(4)	NO_2_	1.324 ***	−275.997 ***	YES	YES	YES	0.666	280,685
			[0.110]	[19.339]					
	(5)	CO	−0.004	−7.962 ***	YES	YES	YES	0.562	280,685
			[0.003]	[0.475]					
	(6)	O_3_	−7.452 ***	304.430 ***	YES	YES	YES	0.847	280,685
			[0.370]	[21.346]					
Other cities	(7)	PM_2.5_	−1.685 ***	−435.336 ***	YES	YES	YES	0.545	202,575
			[0.271]	[26.110]					
	(8)	PM_10_	−2.396 ***	−1184.898 ***	YES	YES	YES	0.613	202,575
			[0.363]	[58.371]					
	(9)	SO_2_	−2.283 ***	−187.511 ***	YES	YES	YES	0.602	202,575
			[0.109]	[9.589]					
	(10)	NO_2_	3.394 ***	−401.190 ***	YES	YES	YES	0.652	202,575
			[0.087]	[21.000]					
	(11)	CO	−0.025 ***	−10.667 ***	YES	YES	YES	0.574	202,575
			[0.004]	[0.531]					
	(12)	O_3_	−8.160 ***	355.084 ***	YES	YES	YES	0.834	202,575
			[0.328]	[20.047]					

Notes: Robust standard errors are clustered at city level in parentheses; *** represent 1% significance levels, respectively.

**Table 5:** Estimation results of the BSDW policy in heating seasons.

Variables	PM_2.5_	PM_10_	SO_2_	NO_2_	CO	O_3_
	(1)	(2)	(3)	(4)	(5)	(6)
time	−27.277 ***	−42.458 ***	−6.337 ***	−16.392 ***	−0.410 ***	7.068 ***
	[1.485]	[2.194]	[0.303]	[0.634]	[0.020]	[0.547]
treat	35.685 ***	37.154 ***	−1.595 **	7.779 ***	0.006	2.640 ***
	[1.709]	[3.028]	[0.750]	[0.987]	[0.029]	[0.620]
time × treat (*β*_3_)	2.099 ***	15.126 ***	−1.316 ***	5.527 ***	−0.023 ***	−8.558 ***
	[0.455]	[0.524]	[0.138]	[0.153]	[0.005]	[0.209]
Controls	YES	YES	YES	YES	YES	YES
Time_FE	YES	YES	YES	YES	YES	YES
City_FE	YES	YES	YES	YES	YES	YES
N	113,640	113,640	113,640	113,640	113,640	113,640
within R^2^	0.591	0.610	0.513	0.643	0.524	0.755

Note: Standard errors are clustered by city level and reported in parentheses; ***, **, represent significant levels of 1%, 5%, respectively.

**Table 6:** Estimation results of the BSDW policy on different cities.

Region		Variables	Time × Treat (*β*_3_)	Controls	Time_FE	City_FE	within R^2^	N
Baoding	(1)	PM_2.5_	−0.530 *** [0.266]	YES	YES	YES	0.645	172,280
	(2)	PM_10_	−1.288 *** [0.482]	YES	YES	YES	0.696	172,280
	(3)	SO_2_	−1.550 *** [0.146]	YES	YES	YES	0.643	172,280
	(4)	NO_2_	1.612 *** [0.147]	YES	YES	YES	0.684	172,280
	(5)	CO	0.004 [0.004]	YES	YES	YES	0.602	172,280
	(6)	O_3_	−6.669 *** [0.585]	YES	YES	YES	0.844	172,280
Beijing	(7)	PM_2.5_	−2.665 *** [0.238]	YES	YES	YES	0.742	163,885
	(8)	PM_10_	−6.327 *** [0.553]	YES	YES	YES	0.774	163,885
	(9)	SO_2_	−0.637 *** [0.093]	YES	YES	YES	0.683	163,885
	(10)	NO_2_	−0.564 *** [0.119]	YES	YES	YES	0.725	163,885
	(11)	CO	0.032 *** [0.004]	YES	YES	YES	0.635	163,885
	(12)	O_3_	−5.435 *** [0.402]	YES	YES	YES	0.863	163,885
Cangzhou	(13)	PM_2.5_	−0.208 [0.156]	YES	YES	YES	0.632	160,600
	(14)	PM_10_	−1.213 *** [0.304]	YES	YES	YES	0.691	160,600
	(15)	SO_2_	−1.912 *** [0.095]	YES	YES	YES	0.645	160,600
	(16)	NO_2_	3.451 *** [0.087]	YES	YES	YES	0.670	160,600
	(17)	CO	−0.009 *** [0.005]	YES	YES	YES	0.586	160,600
	(18)	O_3_	−6.925 *** [0.470]	YES	YES	YES	0.835	160,600
Handan	(19)	PM_2.5_	−4.797 *** [0.416]	YES	YES	YES	0.557	156,950
	(20)	PM_10_	−4.643 *** [0.578]	YES	YES	YES	0.620	156,950
	(21)	SO_2_	−2.549 *** [0.264]	YES	YES	YES	0.552	156,950
	(22)	NO_2_	4.272 *** [0.258]	YES	YES	YES	0.633	156,950
	(23)	CO	0.002 [0.005]	YES	YES	YES	0.522	156,950
	(24)	O_3_	−8.703 *** [0.321]	YES	YES	YES	0.832	156,950
Hengshui	(25)	PM_2.5_	−0.644 *** [0.146]	YES	YES	YES	0.633	152,205
	(26)	PM_10_	−0.538 *** [0.299]	YES	YES	YES	0.681	152,205
	(27)	SO_2_	−2.032 *** [0.091]	YES	YES	YES	0.639	152,205
	(28)	NO_2_	3.722 *** [0.073]	YES	YES	YES	0.668	152,205
	(29)	CO	−0.044 *** [0.006]	YES	YES	YES	0.584	152,205
	(30)	O_3_	−8.618 *** [0.302]	YES	YES	YES	0.840	152,205
Langfang	(31)	PM_2.5_	0.917 *** [0.113]	YES	YES	YES	0.721	145,270
	(32)	PM_10_	−3.814 *** [0.429]	YES	YES	YES	0.760	145,270
	(33)	SO_2_	−1.076 *** [0.118]	YES	YES	YES	0.678	145,270
	(34)	NO_2_	2.121 *** [0.171]	YES	YES	YES	0.701	145,270
	(35)	CO	0.058 *** [0.004]	YES	YES	YES	0.613	145,270
	(36)	O_3_	−8.001 *** [0.331]	YES	YES	YES	0.855	145,270
Tangshan	(37)	PM_2.5_	−0.867 *** [0.205]	YES	YES	YES	0.699	158,410
	(38)	PM_10_	−2.993 *** [0.638]	YES	YES	YES	0.741	158,410
	(39)	SO_2_	−0.799 *** [0.242]	YES	YES	YES	0.591	158,410
	(40)	NO_2_	0.891 *** [0.228]	YES	YES	YES	0.683	158,410
	(41)	CO	−0.043 *** [0.006]	YES	YES	YES	0.560	158,410
	(42)	O_3_	−6.856 *** [0.563]	YES	YES	YES	0.850	158,410
Tianjin	(43)	PM_2.5_	−0.900 *** [0.294]	YES	YES	YES	0.689	155,855
	(44)	PM_10_	−5.414 *** [0.500]	YES	YES	YES	0.740	155,855
	(45)	SO_2_	−0.484 *** [0.156]	YES	YES	YES	0.621	155,855
	(46)	NO_2_	0.463 *** [0.180]	YES	YES	YES	0.680	155,855
	(47)	CO	0.025 *** [0.005]	YES	YES	YES	0.580	155,855
	(48)	O_3_	−5.336 *** [0.505]	YES	YES	YES	0.845	155,855
Xingtai	(49)	PM_2.5_	−5.806 *** [0.406]	YES	YES	YES	0.573	157,315
	(50)	PM_10_	−5.748 *** [0.619]	YES	YES	YES	0.629	157,315
	(51)	SO_2_	−3.212 *** [0.211]	YES	YES	YES	0.593	157,315
	(52)	NO_2_	3.538 *** [0.196]	YES	YES	YES	0.646	157,315
	(53)	CO	−0.053 *** [0.005]	YES	YES	YES	0.560	157,315
	(54)	O_3_	−8.500 *** [0.333]	YES	YES	YES	0.837	157,315
Shijiazhuang	(55)	PM_2.5_	−5.676 *** [0.358]	YES	YES	YES	0.595	161,330
	(56)	PM_10_	−9.215 *** [0.730]	YES	YES	YES	0.649	161,330
	(57)	SO_2_	−4.153 *** [0.226]	YES	YES	YES	0.590	161,330
	(58)	NO_2_	1.144 *** [0.265]	YES	YES	YES	0.666	161,330
	(59)	CO	−0.058 *** [0.006]	YES	YES	YES	0.585	161,330
	(60)	O_3_	−7.870 *** [0.448]	YES	YES	YES	0.840	161,330

Notes: Robust standard errors are clustered at city level in parentheses; *** represent 1% significance levels, respectively.

## Data Availability

The data are not publicly available due to privacy.

## Appendix A

**Table A1:** Air pollution concentration limit of GB3095-2012 and WHO (AQG 2021).

Variables	GB3095-2012		WHO (AQG 2021)				
	The First Level	The Second Level	IT-1	IT-2	IT-3	IT-4	AQG (Air Quality Guidelines)
PM_2.5_	35	75	75	50	37.5	25	15
PM_10_	50	150	150	100	75	50	45
SO_2_	50	150	125	50	-	-	40
NO_2_	80	80	120	50	-	-	25
CO	4	4	7	-	-	-	4
O_3_	100	160	160	120	-	-	100

**Table A2:** Estimation results of the BSDW policy on AQI.

Variables	AQI	
	(1)	(2)
time	−9.128 *** [1.429]	−15.958 *** [1.191]
treat	22.183 *** [1.437]	22.692 *** [1.598]
time × treat (*β*_3_)	−6.060 *** [0.232]	−3.012 *** [0.209]
Controls	NO	YES
Time_FE	YES	YES
City_FE	YES	YES
Constant	70.820 *** [1.415]	−439.295 *** [33.594]
N	345,655	345,655
within R^2^	0.548	0.572

Notes: Robust standard errors are clustered at city level in parentheses; *** represent 1% significance levels, respectively.

**Table A3:** Estimation results of the BSDW policy on different regions.

Region	Beijing			Tianjin			Hebei		
	(1)	(2)	(3)	(4)	(5)	(6)	(7)	(8)	(9)
Variables	PM_2.5_	PM_10_	SO_2_	PM_2.5_	PM_10_	SO_2_	PM_2.5_	PM_10_	SO_2_
time × treat (*β*_3_)	−2.665 *** [0.238]	−6.327 *** [0.553]	−0.637 *** [0.093]	−0.900 *** [0.294]	−5.414 *** [0.500]	−0.484 *** [0.156]	−2.012 *** [0.180]	−3.266 *** [0.334]	−2.261** [0.103]
Constant	−25.844 [35.817]	−400.744 *** [41.695]	−141.209 *** [6.175]	−186.860 *** [26.215]	−592.878 *** [41.935]	−139.454 *** [7.547]	−468.321 *** [31.204]	−1269.380 *** [57.428]	−227.284 *** [9.438]
Controls	YES	YES	YES	YES	YES	YES	YES	YES	YES
Time_FE	YES	YES	YES	YES	YES	YES	YES	YES	YES
City_FE	YES	YES	YES	YES	YES	YES	YES	YES	YES
within R^2^	0.742	0.773	0.682	0.689	0.740	0.621	0.573	0.623	0.572
N	163,885	163,885	163,885	155,855	155,855	155,855	301,125	301,125	301,125

Notes: Robust standard errors are clustered at city level in parentheses; **, *** represent 5%, and 1% significance levels, respectively.

## Appendix B

**Step 1.**

Compare the graded concentration limits of various pollutants (the concentration limits of AQI refer to GB3095-2012, and the concentration limits of API refer to GB3095-1996) and take the measured concentration values of PM_2.5_, PM_10_, SO_2_, NO_2_, CO, O_3_ and other pollutants (PM_2.5_ and PM_10_ were 24 h average concentrations) to calculate the individual air quality index (IAQI), respectively.

(A1) $$IAQI_{P}=\frac{IAQI_{Hi}-IAQI_{L0}}{BP_{Hi}-BP_{L0}}(C_{P}-BP_{L0})+IAQI_{L0}$$

In Formula (A1):

*IAQI_P_*: Air quality sub index of pollutant *p*;

*C_P_*: Mass concentration value of pollutant *p*;

*BP_Hi_*: High value of pollutant concentration limit similar to *C_P_* in Table A4;

*BP*_*L*0_: Lower limit values of pollutant concentration similar to *C_P_* in Table A4;

*IAQI_Hi_*: Air quality sub indices corresponding to *BP_Hi_* in Table A4;

*IAQI*_*L*0_: Air quality sub indices corresponding to *BP*_*L*0_ in Table A4.

**Table A4:** Air quality sub index of corresponding areas and corresponding pollutant project concentration index table.

IAQI	Pollutant Concentration Limit									
	SO_2_ (24 h Average Concentration) (μg/m^3^)	SO_2_ (1 h Average Concentration) (μg/m^3^)	NO_2_ (24 h Average Concentration) (μg/m^3^)	NO_2_ (1 h Average Concentration) (μg/m^3^)	PM_10_ (24 h Average Concentration) (μg/m^3^)	CO (24 h Average Concentration) (mg/m^3^)	CO (1 h Average Concentration) (mg/m^3^)	O_3_ (1 h Average Concentration) (μg/m^3^)	O_3_ (8 h Moving Average) (μg/m^3^)	PM_2.5_ (24 h Average Concentration) (μg/m^3^)
0	0	0	0	0	0	0	0	0	0	0
50	50	150	40	100	50	2	5	160	100	35
100	150	500	80	200	150	4	10	200	160	75
150	475	650	180	700	250	14	35	300	215	115
200	800	800	280	1200	350	24	60	400	265	150
300	1600	(2)	565	2340	420	36	90	800	800	250
400	2100	(2)	750	3090	500	48	120	1000	(3)	350
500	2620	(2)	940	3840	600	60	150	1200	(3)	500

**Step 2.**

The maximum value of IAQI of each pollutant is determined as AQI. When AQI is greater than 50, the pollutant with the largest IAQI is determined as the primary pollutant.

AQI = max{IAQI_1_, IAQI_2_, IAQI_3_, …, IAQI_n_} (A2)

In Formula (A2):

IAQI: Air quality sub index;

n: Air pollutant.
